# microRNA-3129 promotes cell proliferation in gastric cancer cell line SGC7901 via positive regulation of pRb

**DOI:** 10.1590/1414-431X20186452

**Published:** 2018-05-21

**Authors:** Shaofeng Yang, Nan Sheng, Lili Pan, Jing Cao, Jiao Liu, Ran Ma

**Affiliations:** Department of Gastroenterology, Jining No. 1 People’s Hospital, Jining, China

**Keywords:** microRNA-3129, Gastric cancer, pRb, Proliferation, Cell cycle

## Abstract

Several microRNAs (miRNAs) have been reported as oncogenes or tumor suppressors in many cancers, including gastric cancer (GC). However, the role and molecular mechanism of miR-3129 in GC is largely unknown. We aimed to explore the function and the underlying molecular mechanism of miR-3129 in GC. Cancer tissues and corresponding adjacent tissues were collected from 50 patients with GC, and the expression of miR-3129 was detected by RT-qPCR. The expression of miR-3129 and pRb in human GC cell line SCG7091 was altered by transient transfection. Thereafter, MTT and flow cytometry assays were used to analyze cell viability and cell cycle. The expression of cyclin E, CDK2, CDK2 inhibitors (p16 and 21), and pRb were detected by RT-qPCR and western blot. A significant up-regulation of miR-3129 was observed in GC tissues compared to adjacent tissues. Overexpression of miR-3129 significantly improved cell viability after 4 days of post-transfection. Flow cytometry assay results showed that the miR-3129 overexpression arrested more SGC7901 cells at S phase. Moreover, overexpression of miR-3129 down-regulated the expression of CDK2 inhibitors while it up-regulated the expression levels of cyclin E, CDK2, and pRb. Interestingly, we found that pRb inhibition reversed the effect of miR-3129 inhibitor on cell proliferation in SGC7901 cells, increased cell viability, reduced cells at G0/1 phase, and modulated the expression of proliferation-related factors. Our results revealed that miR-3129 functioned as an oncogene through positive regulation of pRb and may prove to be a promising option for molecular therapy of GC.

## Introduction

Gastric cancer (GC) is one of the most common malignancies and the second leading cause of cancer-related death worldwide (1). According to the results of 2016 cancer estimation, there were 26,370 new GC cases and 10,730 deaths occurred in the USA. The highest incidence rate was concentrated in Eastern Asia, Central and Eastern Europe, and South America (2). Currently, treatment options in the adjuvant therapy of GC remain limited due to the poor early diagnosis and prognosis. Therefore, to find and develop promising biomarkers of screening for GC is urgently needed (3). An increasing number of studies have revealed that microRNAs (miRNAs) are deregulated in a variety of malignancies and play a critical role in the development and process of tumors. Thus, exploring the role of miRNAs in tissues or cells may provide the basis for their development as novel diagnostic, prognostic, and predictive biomarkers, as well as therapeutic targets (4).

miRNAs are short (20–24 nt) non-coding RNAs involved in post-transcriptional regulation of gene expression in multicellular organisms by affecting both the stability and translation of mRNAs (5). Human genome research has confirmed that at least 200 miRNAs are associated with a number of cancer types, including GC (6). For instance, miR-9, miR-421, miR-27a, and miR-143 have been reported to be involved in the development and progression of the GC by regulating tumor cells proliferation, apoptosis, invasion, and metastasis (7–11). Regarding miR-3129, previous studies have demonstrated that it might be associated with the risk of cancers including colorectal and breast cancer (12,13). However, the role of miR-3129 in GC has not been demonstrated yet.

The retinoblastoma protein (pRb) is encoded by the RB1 gene, which has been identified as a tumor suppressor protein and as dysfunctional in several cancers (14). pRb plays key roles in cell cycle regulation through its ability to bind and interact with a variety of cellular proteins, which is governed by its phosphorylation state (15). When pRb is bound to E2F, the interactive complex acts as a growth suppressor and prevents progression by controlling cell cycle (16). Evidence has proven that pRb is capable of controlling cell cycle by E2F-independent effects (17). Nevertheless, whether miR-3129 could alter pRb and thus further participate in the development and progression of GC has not been reported.

Therefore, we aimed to investigate the role of miR-3129 in GC cells and explore the underlying molecular mechanisms. Our results might provide a new insight into the potential molecular therapy for GC.

## Material and Methods

### Patient samples

GC tissues and corresponding adjacent tissues from 50 patients (29 males and 21 females, aged from 30–66 years) who underwent resection of primary GC were used. The samples were collected in Jining No. 1 People’s Hospital between February 2015 and August 2016, and stored at -80°C. Patients had not received radiotherapy or chemotherapy prior to surgery. Tumor grade was determined according to various classifications of tumors (18). Eleven cases were well differentiated, ten moderately differentiated, fifteen poorly, and fourteen undifferentiated by pathological grading. GC patients were classified into four stages according to TNM classification. Twelve cases were in TNM stage I, nineteen in TNM stage II, nine in TNM stage III, and ten in TNM stage IV (Table 1). The study was approved by the Research Ethics Committee of Jining No. 1 People’s Hospital and written informed consent was obtained from all patients.

**Table 1.** Clinicopathological features of 50 patients with gastric cancer.

Parameters	N	P value (chi-squared test)
Age (years)		0.749
<50	22	
>50	28	
Gender		0.704
Male	29	
Female	21	
Size		0.665
>3cm	19	
≤3cm	31	
Differentiation			0.815
Well	11	
Moderately	10	
Poorly	15	
Undifferentiated	14	
TNM stage			0.897
I	12	
II	19	
III	9	
IV	10	

TNM: tumor, node, metastasis.

### Cell culture

Human GC cell line SGC7901 was purchased from Ambion (Invitrogen, USA). Cells were cultured in Roswell Park Memorial Institute (RPMI)-1640 medium (Invitrogen) supplemented with 10% fetal bovine serum (FBS; Gibco, USA), 100 U/mL of penicillin sodium, and 100 µg/mL streptomycin sulfate (Gibco) in a humidified atmosphere with 5% CO_2_ at 37°C (19).

### Cell transfection

Synthetic miR-3129 mimic, miR-3129 inhibitor, and scrambled control RNA were purchased from Genepharma (China). The scrambled control RNA represents a universal control for both inhibitors and mimics that are based on the sequences of miRNAs in *C. elegans*. SGC7901 cells were seeded in 6-well plates and transfected with miR-3129 mimic, miR-3129 inhibitor, and control using Lipofectamine 2000 (Invitrogen) on the following day when the cells were approximately 70% confluent. In each well, equal amounts (100 pmol) of miR-3129 mimic, inhibitor, and control were used (20).

For the silencing of pRb, siRNA targeted pRb (si-pRb) or the corresponding negative control (si-NC) was synthesized by Genepharma. SGC7901 cells were seeded onto 6-well plates and then transfected with si-NC or si-pRb by using Lipofectamine 2000 (Invitrogen) when the confluence was reached to approximately 70%. After transfection for 48 h, cells were harvested and then used for the subsequent experiments.

### Cell viability assay

Cell viability was assessed using the 3-(4,5-dimethylthiazol-2-yl)-2,5-diphenyl tetrazolium bromide (MTT) colorimetric assay. Briefly, 100-μL transfected-cells suspension were seeded onto 96-well plate and incubated for 1–5 days. Subsequently, 50 μL of MTT (Beyotime, China) was added to culture medium and this mix was incubated for another 4 h at 37°C. Thereafter, 100 μL solution of 4% HCl 1N in 2-propanol was mixed thoroughly into each well. Plates were read on a microplate reader (Molecular Devices, USA) at a wavelength of 570 nm, with a background reading at 650 nm subtracted. Triplicate readings for each sample were averaged (21).

### Cell cycle analysis by flow cytometry

Cell cycle analysis is a method that employs flow cytometry to distinguish cells from different phases of the cell cycle. Briefly, SGC-7901 cells were plated in 96-well plates and transfected with miR-3129 mimic, miR-3129 inhibitor or control for 48 h at 37°C. Cells were harvested at 48 h post-transfection, washed once with phosphate-buffered saline (PBS), and then fixed overnight in 70% ethanol at 4°C and stained for DNA content using PI (50 μg/mL) with RNase A (100 μg/mL) (Beyotime, China), followed by incubation for 30 min in the dark at room temperature. The percentage of the cells in G0/G1, S, and G2/M phases were counted and compared using a FACScan flow cytometer (Becton Dickinson, USA) (22).

### RNA extraction and quantitative real-time PCR (RT-qPCR) analysis

Total RNAs of SGC7901 cells and patient samples were extracted with TRIzol reagent (Invitrogen) according to manufacturer's protocol. First strand complimentary DNA (cDNA) was synthesized using PrimeScript 1^st^ Strand cDNA Synthesis Kit (Invitrogen) following the manufacturer's instructions. RT-qPCR was performed using an Applied Biosystems 7900 Real-time PCR system and a Fast Start Universal SYBR green Master (Roche) with the universal reverse primer provided in the kit. The thermocycling parameters were 95°C for 3 min and 40 cycles of 95°C for 15 s followed by 60°C for 30 s. Each sample was run in triplicate and was normalized to U6 snRNA level (forward: 5′-CTT CGG CAG CAC ATA TAC T-3′ and reverse: 5′-AAA ATA TGG AAC GCT TCA CG-3′). The specific primer for miR-3129 was forward: 5′-GGG GCA GTA GTG TAG AGA T-3′ and reverse: 5′-CAG TGC GTG TCG TGG AGT-3′. Melting curve analysis was performed to confirm the specificity of the PCR products. The replicates were then averaged, and fold induction was determined by a ΔΔCT-based fold change calculation (23).

### Western blot analysis

The cells were washed twice with PBS and then lysed with 1× sodium dodecyl sulfate (SDS)-loading buffer (50 mM Tris-Cl, pH 6.8, 100 mM DTT, 2% SDS, 10% glycerol, and 0.1% bromophenol blue) as the whole-cell sample. The protein samples were subjected to SDS-polyacrylamide gel electrophoresis (SDS-PAGE). Immunoblottings were carried out with primary antibodies cyclin E (ab3927), cyclin-dependent kinase 2 (CDK2; ab6433), β-actin (ab8227) (Abcam, UK), and pRb (#9313; Cell Signaling Technology, USA). After being washed three times with PBS, the membranes were incubated in horseradish peroxidase-conjugated secondary antibodies at room temperature for 1 h. Proteins bands were developed by enhanced chemiluminescence (ECL-plus, Amersham Pharmacia Biotech) and visualized by using Image Lab™ Software (Bio-Rad, USA).

### Statistical analysis

All experiments were repeated independently three times. Data are reported as means±SD of multiple experiments. Each set of data was tested for normal distribution using the Kolmogorov-Smirnov test as well as for homogeneity of variances prior to statistical analysis. Statistical analyses were performed using the GraphPad Prism 5.0 software (GraphPad Software, USA) by the chi-squared test for clinicopathological features, Student's *t*-test for comparisons of two groups, and one-way ANOVA followed by Tukey *post hoc* test for comparisons of three groups or more. P<0.05 was considered statistically significant.

## Results

### Expression of miR-3129 was up-regulated in GC tissues

**Figure 1.**
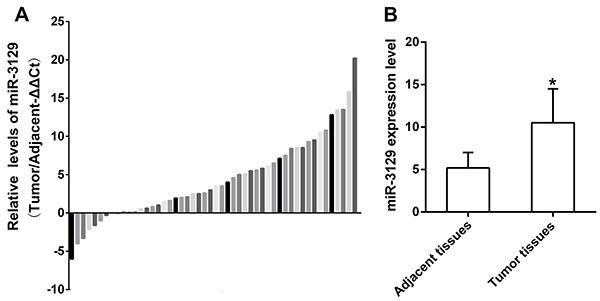
Relative miR-3129 expression in human gastric cancer (GC) tissues. *A*, Relative expression of miR-3129 in GC tissues when compared with adjacent tissues, which were detected by RT-qPCR. *B*, Relative expression of miR-3129 in tumor and adjacent tissues. RT-qPCR: quantitative real time polymerase chain reaction. Data are reported as means±SD. *P<0.05 (*t*-test).

There was no significant difference in clinicopathological features such as age, gender, tumor size, level of differentiation, and TNM stage of 50 patients (Table 1). RT-qPCR results showed that, among 50 patients, 41 (82%) presented highly expressed miR-3129, while miR-3129 was down-regulated in 9 (18%) GC patients (Figure 1A). In addition, results in Figure 1B showed that miR-3129 expression level was significantly higher in tumor tissues than adjacent tissues (P<0.05), implying miR-3129 might be related to GC. Therefore, we analyzed its roles in SGC7901 cells in the following experiments.

### miR-3129 promoted cell viability of SGC7901 cells

**Figure 2.**
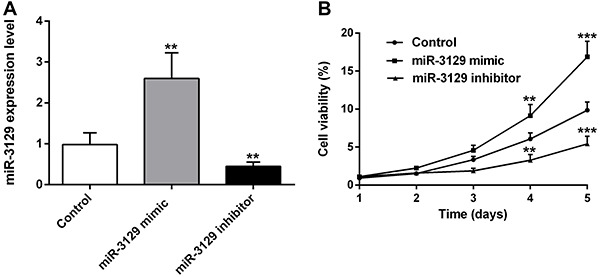
Effect of miR-3129 expression on SGC7901 cells viability. *A*, miR-3129 mimic and inhibitor were firstly transfected into SGC7901 cells to inhibit and overexpress miR-3129 expression by RT-qPCR. *B*, MTT assay was performed to determine SGC-7901 cells viability after the transfected cells were cultured for 1–5 days. RT-qPCR: quantitative real time polymerase chain reaction. Data are reported as means±SD. **P<0.01, ***P<0.001 (ANOVA followed by Tukey *post hoc* test).

To investigate the functional role of miR-3129 in GC cells, miR-3129 mimic and inhibitor were transfected into SGC7901 cells to suppress and overexpress, respectively, miR-3129 levels. RT-qPCR assays revealed that miR-3129 expression was significantly promoted by miR-3129 mimic in SGC-7901 (P<0.01), and reduced by miR-3129 inhibitor (P<0.01) (Figure 2A). To assess the regulatory effect of miR-3129 on cell viability, MTT assay was performed at 1, 2, 3, 4, and 5 days after miR-3129 transfection. As shown in Figure 2B, miR-3129 overexpression significantly increased cell viability of GC cells at 4 days post-transfection (P<0.01 or P<0.001). On the contrary, miR-3129 inhibition dramatically reduced cell viability at 4 days post-transfection in GC cells (P<0.01 or P<0.001). These results indicated that miR-3129 acted as an oncogene by improving GC cells viability.

### miR-3129 induced S phase arrest in SGC7901 cells

**Figure 3.**
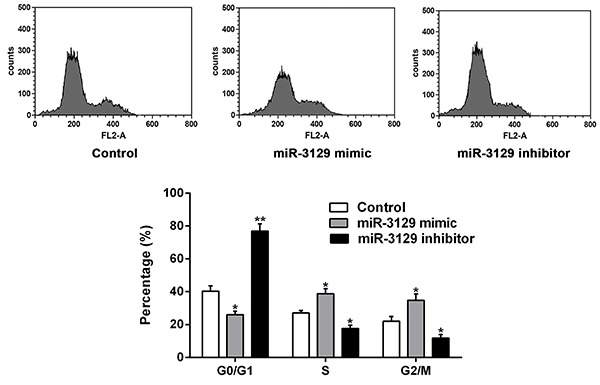
Effect of miR-3129 on gastric cancer cell cycle. After transfection with miR-3129 mimic and inhibitor, the percentage of cells in G1/G0, S, and G2/M phases was analyzed by flow cytometry. Data are reported as means±SD. *P<0.05, **P<0.01 (ANOVA followed by Tukey *post hoc* test).

We further examined the effect of miR-3129 on cell proliferation of GC cells through using flow cytometry. miR-3129 mimic significantly reduced the rates of cell at G0/G1 phase but increased the number of cells at S and G2/M phases (Figure 3; P<0.05). A completely opposite result was observed in the regulation of miR-3129 inhibition on cell cycle (P<0.05 or P<0.01). These results indicated that miR-3129 overexpression in SGC-7901 induced cell cycle arrest at S phase.

### miR-3129 improved the expression of cyclin E and CDK2 in SGC7901 cells

**Figure 4.**
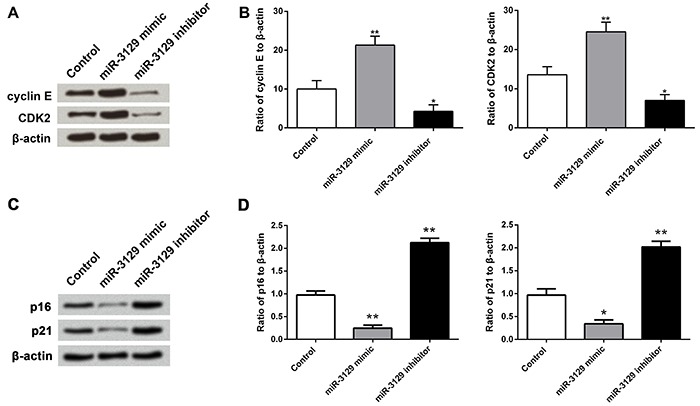
Effects of miR-3129 on cyclin E and CDK2 expression in SGC7901 miR-transfected cells. *A*, Western blotting was performed to assess the expression of cyclin E and CDK2. *B*, RT-qPCR was performed to assess the expression of cyclin E and CDK2. *C*, Western blotting was performed to assess the expression of p16 and 21. *D*, RT-qPCR was performed to assess the expression of p16 and 21. CDK: cyclin dependent kinase; RT-qPCR: quantitative real time polymerase chain reaction. Data are reported as means±SD. *P<0.05, **P<0.01 (ANOVA followed by Tukey *post hoc* test).

Cyclin E and CDK2 are two vital regulators of cell cycle. CDK2 can form complexes with cyclins and be activated in the late G1 phase, and thus promote G1/S transition (24). Therefore, these two factors were used to verify the function of miR-3129 on cell cycle. Western blotting results showed that compared with the control group, the expression of cyclin E and CDK2 were both up-regulated by miR-3129 mimic but down-regulated by miR-3129 inhibitor (Figure 4A). Similar results were observed in the mRNA expression by RT-qPCR analysis, as miR-3129 overexpression significantly increased the mRNA levels of cyclin E and CDK2 (P<0.01), while miR-3129 inhibition reduced the mRNA expressions of both factors (P<0.05) (Figure 4B). We also investigated the effect of miR-3129 on the expression of CDK inhibitors including p16 and p21. As shown in Figure 4C, the expressions of p16 and p21 were both inhibited by miR-3129 mimic but enhanced by miR-3129 inhibitor. Consistently, the mRNA levels of p16 and p21 were down-regulated by miR-3129 mimic while up-regulated by miR-3129 inhibitor (P<0.05 or P<0.01) (Figure 4D). These data suggested that miR-3129 overexpression was able to modulate SGC7901 cells cycle via regulation of cyclin E and CDK2.

### miR-3129 regulated pRb in SGC7901 cells

**Figure 5.**
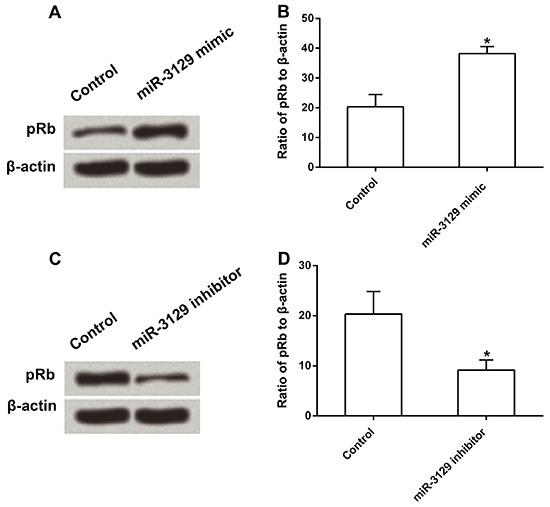
Effects of miR-3129 on pRb expression in SGC7901 cells. *A*, Western blot and *B*, RT-qPCR analyses were performed to determine the levels of pRb in miR-3129-overexpressing cells. *C*, Western blot and *D*, RT-qPCR analyses were performed to determine the levels of pRb in miR-3129-suppressing cells. pRb: retinoblastoma protein; RT-qPCR: quantitative real time polymerase chain reaction. Data are reported as means±SD. *P<0.05 (Student's *t*-test).

Previous studies have indicated the important roles of pRb in the cell cycle (25). We further investigated the effects of miR-3129 on SGC-7901 cell cycle by detecting pRb expression. Western blot and RT-qPCR analytical results showed that the expression of pRb was significantly up-regulated in miR-3129-overexpressing cells (P<0.05) (Figure 5A and B), while pRb was obviously down-regulated in miR-3129-suppression cells (P<0.05) (Figure 5C and D). Thus, we inferred that miR-3129 could regulate pRb expression in SGC7901 cells.

### pRb silencing reversed the effect of miR-3129 inhibitor on cell proliferation of SGC7901 cells

**Figure 6.**
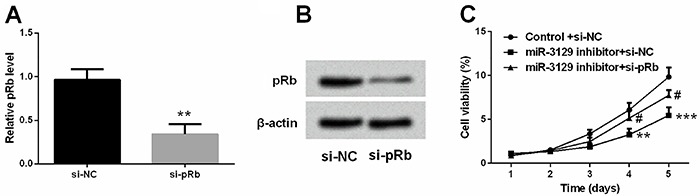
Effects of si-pRb transfection on pRb expression and cell viability of SGC7901 cells transfected with si-pRb and si-NC for 48 h. *A*, RT-qPCR and *B*, western blot analysis was performed to assess the expression levels of pRb. SGC7901 cells were co-transfected with miR-3129 inhibitor and si-NC, or co-transfected with miR-3129 inhibitor and si-pRb. *C*, cell viability was measured by MTT assay. RT-qPCR: quantitative real time polymerase chain reaction; pRb: retinoblastoma protein; si-pRb: siRNA targeted pRb. Data are reported as means±SD. **P<0.01, ***P<0.001, ^#^P<0.05 (ANOVA followed by Tukey *post hoc* test).

To further investigate whether miR-3129 had a role in cell proliferation of SGC7901 cells through targeting pRb, the expression of pRb in SGC7901 cells was knockdown by transfection with si-pRb. The transfected efficiency was identified by RT-qPCR and western blot. As expected, the mRNA and protein expression of pRb was efficiently suppressed by si-pRb transfection (Figure 6A and B, P<0.01). Then, SGC7901 cells were co-transfected with si-pRb and miR-3129 inhibitor, and cell viability, cell cycle, and expression levels of proliferation-related factors were measured. We found that knockdown of pRb reversed the effect of miR-3129 inhibitor on cell viability, as the co-transfection of miR-3129 inhibitor and si-pRb significantly increased cell viability of SGC7901 cells, compared with co-transfection with miR-3129 inhibitor and si-NC (Figure 6C, P<0.05, P<0.01 or P<0.001).

**Figure 7.**
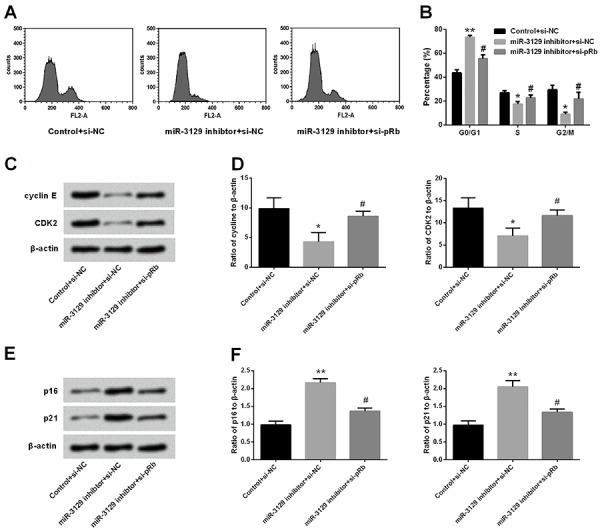
Effects of pRb silencing on cell cycle arrest and expression levels of proliferation-related factors. SGC7901 cells were co-transfected with miR-3129 inhibitor and si-NC, or co-transfected with miR-3129 inhibitor and si-pRb, then, *A* and *B*, the ratio of cells at G0/1, S, and G2/M phases was analyzed by flow cytometry. *C*, Western blot analysis and *D*, RT-qPCR were performed to determine the protein and mRNA expression of cyclin E and CDK2 in SGC7901 cells. *E*, Western blot analysis and *F*, RT-qPCR were performed to determine the protein and mRNA expression of p16 and 21 in SGC7901 cells. RT-qPCR: quantitative real time polymerase chain reaction; pRb: retinoblastoma protein; si-pRb: siRNA targeted pRb; CDK: cyclin dependent kinase. Data are reported as means±SD. *P<0.05, **P<0.01, ^#^P<0.05 (ANOVA followed by Tukey *post hoc* test).

Similarly, we also found that pRb silencing reversed the effect of miR-3129 inhibitor on cell cycle arrest and the expression of proliferation-related factors. As shown in Figure 7A and B, co-transfection of miR-3129 and si-pRb significantly increased the rates of cells at S and G2/M phases but reduced the rate of cells at G0/G1 phase, compared with co-transfection with miR-3129 inhibitor and si-NC. Meanwhile, the silencing of pRb efficiently abolished the effect of miR-3129 on the expression of proliferation-related factors, as pRb inhibition significantly up-regulated the expression of cyclin E and CDK2, but down-regulated the expression of p16 and p21 in miR-3129-transfected cells (Figure 7C-F, P<0.05 or P<0.01). Taken together, these results indicated that pRb mediated the modulatory effect of miR-3129 on cell proliferation of SGC7901 cells.

## Discussion

Mortality remains high in GC patients due to the lack of effective methods of diagnosis and treatment (26). Since the discovery of the miRNA, it became clear that various carcinomas have specific miRNA expression patterns (27); however, little has been found about miR-3129 action in GC. Our study first reported the functional roles of miR-3129 in GC showing it was significantly up-regulated in GC tissues and promoted the proliferation of SGC7901 cells. Moreover, overexpression of miR-3129 up-regulated the expression levels of cyclin E, CDK2, and pRb.

Accumulating evidence has highlighted the aberrant expressions of miRNA and the important regulatory roles of miRNAs in GC. For example, miR-221-3p (28), miR-421 (8 [Bibr B09] [Bibr B10]), miR-21 (27), miR-150 (20), miR-199a (3), and miR-106b (29) have been reported to be up-regulated in GC. In addition, miR-509-3p (30), miR-375 (31), miR-9 (7), miR-101, and miR-128 (32) are down-regulated. In the present study, we found that miR-3129 was significantly up-regulated in GC tissues for the first time, implying it might be related to the progression of GC.

Tumor cells are characterized by accelerated proliferation and uncontrolled growth. Many studies demonstrated that some miRNAs such as miR-199a (3), miR-27b (32), and miR-150 (20) could regulate tumor cells proliferation. However, to our knowledge, few studies have reported the association between miR-3129 and GC. In our study, we found that overexpression of miR-3129 in SGC7901 cells could efficiently promote cell viability, suggesting that it might act as an oncogene that could regulate the cell proliferation of GC cells.

At present, many studies have illustrated the inhibition of tumor cell proliferation by arresting cells at different phases, which may provide therapeutic benefit against the tumorigenesis (22). Xiong et al. (33) demonstrated that the down-regulation of miR-214 could induce G1 cell cycle arrest in GC. Furthermore, Zhao et al. found that miR-638 overexpression and Sp2-siRNA markedly suppressed cell proliferation with decreasing expression of cyclin D1 and induced G1 phase cell cycle arrest *in vitro* (34). Our data also suggested that miR-3129 could promote GC cells proliferation by arresting more cells at S phase.

Research on pRb shows that it plays a fundamental role in cell cycle mechanisms (35). pRb is obtained when the Rb is phosphorylated by CDKs (36), then it is able to release E2F. E2F is a transcription factor, which could promote the transcription of cyclin E and CDK protein and enhance the rate of cells in S phase (37). That is, the completion of the cell cycle requires the assistance of CDKs and cyclin E. In GC, studies have shown that miRNAs could regulate cell cycle via modulating CDKs, cyclin E, and pRb thereby driving cell proliferation (38). Our study was consistent with previous studies showing that miR-3129 could positively regulate the levels of pRb, cyclin E, and CDK2. Thus, we further explored whether miR-3129 possessed its role through regulation of pRb. Interestingly, we found that pRb silencing abolished the effect of miR-3129 inhibitor on cell proliferation in SGC7901 cells, implying that pRb mediated the role of miR-3129 in SGC7901 cells. However, further investigations need to discover the underlying mechanisms of the novel therapeutic strategies.

In summary, these results provide new evidence confirming that miR-3129 functioned as an oncogene in GC. miR-3129 promoted GC cell proliferation and cell cycle progression by positively modulating the expression of pRb. miR-3129 might be a candidate predictor or an anticancer therapeutic target for GC patients.
